# Virtual brain twins: from basic neuroscience to clinical use

**DOI:** 10.1093/nsr/nwae079

**Published:** 2024-02-28

**Authors:** Huifang E Wang, Paul Triebkorn, Martin Breyton, Borana Dollomaja, Jean-Didier Lemarechal, Spase Petkoski, Pierpaolo Sorrentino, Damien Depannemaecker, Meysam Hashemi, Viktor K Jirsa

**Affiliations:** Aix Marseille Université, Institut National de la Santé et de la Recherche Médicale, Institut de Neurosciences des Systèmes (INS) UMR1106; Marseille 13005, France; Aix Marseille Université, Institut National de la Santé et de la Recherche Médicale, Institut de Neurosciences des Systèmes (INS) UMR1106; Marseille 13005, France; Aix Marseille Université, Institut National de la Santé et de la Recherche Médicale, Institut de Neurosciences des Systèmes (INS) UMR1106; Marseille 13005, France; Service de Pharmacologie Clinique et Pharmacosurveillance, AP–HM, Marseille, 13005, France; Aix Marseille Université, Institut National de la Santé et de la Recherche Médicale, Institut de Neurosciences des Systèmes (INS) UMR1106; Marseille 13005, France; Aix Marseille Université, Institut National de la Santé et de la Recherche Médicale, Institut de Neurosciences des Systèmes (INS) UMR1106; Marseille 13005, France; Aix Marseille Université, Institut National de la Santé et de la Recherche Médicale, Institut de Neurosciences des Systèmes (INS) UMR1106; Marseille 13005, France; Aix Marseille Université, Institut National de la Santé et de la Recherche Médicale, Institut de Neurosciences des Systèmes (INS) UMR1106; Marseille 13005, France; Aix Marseille Université, Institut National de la Santé et de la Recherche Médicale, Institut de Neurosciences des Systèmes (INS) UMR1106; Marseille 13005, France; Aix Marseille Université, Institut National de la Santé et de la Recherche Médicale, Institut de Neurosciences des Systèmes (INS) UMR1106; Marseille 13005, France; Aix Marseille Université, Institut National de la Santé et de la Recherche Médicale, Institut de Neurosciences des Systèmes (INS) UMR1106; Marseille 13005, France

**Keywords:** virtual brain twin, personalized modeling, inference, neuroscience, brain disorder

## Abstract

Virtual brain twins are personalized, generative and adaptive brain models based on data from an individual’s brain for scientific and clinical use. After a description of the key elements of virtual brain twins, we present the standard model for personalized whole-brain network models. The personalization is accomplished using a subject’s brain imaging data by three means: (1) assemble cortical and subcortical areas in the subject-specific brain space; (2) directly map connectivity into the brain models, which can be generalized to other parameters; and (3) estimate relevant parameters through model inversion, typically using probabilistic machine learning. We present the use of personalized whole-brain network models in healthy ageing and five clinical diseases: epilepsy, Alzheimer’s disease, multiple sclerosis, Parkinson’s disease and psychiatric disorders. Specifically, we introduce spatial masks for relevant parameters and demonstrate their use based on the physiological and pathophysiological hypotheses. Finally, we pinpoint the key challenges and future directions.

## Teaser

Virtual brain twins integrate individual brain imaging data in a digital model to accurately reflect a person's brain. This review explains how to use them for various brain diseases.

## VIRTUAL BRAIN TWINS

A virtual brain twin is a special case of a ‘digital twin’, which originated in the realm of industry [1,2], and is a personalized, generative and adaptive brain model, adequately representing an individual’s brain at the system level of description. The model is informed by subject-specific data, and aims to guide decision making in diagnostics, prognosis and therapy. The aim is thus not to resemble a biological brain in as much detail as possible, but rather to be able to mechanistically explain and capture the most relevant data features, answering a specific research or clinical question. In other words, one wishes to keep the individual’s brain twin as simple as possible, but as complex as necessary [3].

Several review papers on digital twins in healthcare and various brain disorders [4–6] have offered high-level descriptions of concepts and technologies. In this paper, we present a more formalized and comprehensive conceptual framework as the starting point for virtual brain twins. Specifically, we provide a formal definition of a virtual brain twin and its key elements, propose a unified framework from the perspective of personalized whole-brain network modeling and deliver concrete examples for different clinical applications.

**Figure 1.**
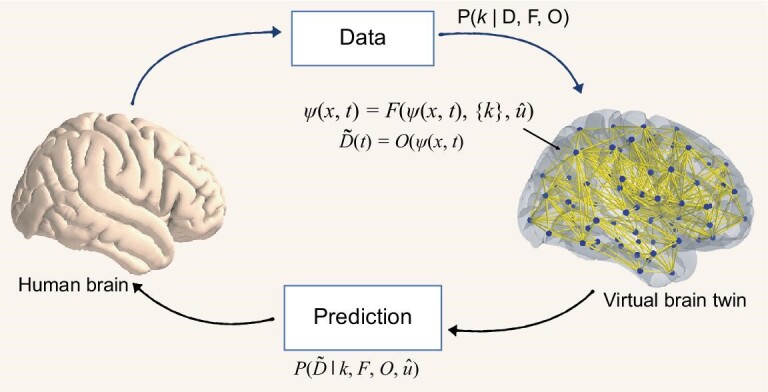
The key elements of virtual brain twins. The brain activity, denoted ψ at position *x* at time *t*, of virtual brain twins can be computed using model *F* and the set of control parameters {*k*}. Simulated brain activity data are mapped on sensor data $\tilde{D}(t)$ through the forward solution *O*. We map real-world data *D*, observed in the human brain, onto the space of the virtual brain and personalize its control parameters *k*. Clinical interventions $\hat{u}$ represent any external operation capable of influencing the brain dynamics. Virtual brain twins generate predictions by simulating $\tilde{D}(t)$ under various conditions.

Figure 1 illustrates a human brain and its virtual brain twin, as well as their relationships. From a human brain, we can obtain multimodal data, denoted *D*. These data might be anatomical such as T1-weighted MRI (T1-MRI), diffusion-weighted MRI (DW-MRI), computed tomography (CT) and positron emission tomography (PET) scans; functional such as electroencephalogram (EEG), magnetoencephalography (MEG), stereo EEG (SEEG) and functional MRI (fMRI); or of other types such as demographics, genetics and behavioral data. These multimodal data are typically integrated into a personalized model to perform informed predictions about brain function. The equations of a virtual brain twin are


(1a) \begin{eqnarray*}
\dot{\psi }(x,t) = F(\psi (x,t),\lbrace k\rbrace ,\hat{u}),
\end{eqnarray*}

(1b) \begin{eqnarray*}
\tilde{D}(t) = O(\psi (x,t)).
\end{eqnarray*}

Equation 1a prescribes the evolution of neural activity in time and in brain space. Equation 1b describes how the recorded signal derives from current neural activity. The separation into generative brain dynamics (Equation 1a) and observer (Equation 1b) is well established in full brain network modeling [7] and inference frameworks such as dynamical causal modeling [8]. Let ψ(*x, t*) be the neural activity at time *t* and position *x*. The dot placed above ψ(*x, t*) indicates its first derivative with respect to time. Here *F* describes the neural field as a function of neural activity ψ(*x, t*), a set of control parameters {*k*} and clinical interventions $\hat{u}$. Estimated $\tilde{D}(t)$ can be calculated from forward solution *O* as a function of brain activity ψ(*x, t*). The set of control parameters {*k*} is a subset of all model parameters and, in the context of this article, is specific and causal to a disease and the healthy ageing process. Each control parameter *k* can be derived from its posterior distribution given the observed or recorded data *D*, the brain dynamic model *F* and the forward solution *O*, i.e. *P*(*k*∣*D, F, O*). The virtual brain twin is predictive for individual patients by generating simulated data $\tilde{D}(t)$ using the patient-specific framework described by {*k*}, *F, O* and $\hat{u}$, which captures inter-subject variability, disease specificity, sensor placements and clinical interventions.

Mathematically, the intervention $\hat{u}$ can represent different operations, as indicated by the hat operator, influencing the brain dynamics, such as stimulation [9], surgical intervention, medication effects and even lifestyle change. For instance, $\hat{u}$ may correspond to therapeutic electric stimulation in epilepsy, given certain stimulation parameter settings such as location of electrodes, stimulation frequency and amplitude. Given an objective function, we can then optimize a set *U* of the $\hat{u}$ for the patient’s brain model and transfer the solutions with preferred outcome as a recommendation to the real world. Note that while the virtual brain twin can generate functional signals resembling those of the human brain, the external intervention $\hat{u}$ is not just a simple replication, owing to the intricate interaction between biological, physical and the model processes [10]. We can further adapt the personalized model in the next iteration of the virtual brain twin loop. If $\hat{u}$ has qualitatively changed the physical brain responses, we can improve and adapt the virtual brain using the new recordings of the physical brain.

### Standard model of the virtual brain twin

We introduce the term ‘standard model’ in reference to a generic virtual brain model, which serves as a starting point for the process of personalization. The standard model integrates the various concepts and methods of virtual brain modeling of the past 20 years in the same modular framework, providing a large degree of adaptability and emphasizing methods of neural mass and neural field large-scale modeling [11–15], nonlinear dynamics [16,17] and network science [18–21]. As a starting point, we expand the neural activity Equation 1a into the Jirsa–Haken equation comprising its three components, that is, local node dynamics, and local and global network interactions [22,23], establishing the standard model as follows:


(2) \begin{eqnarray*}
&& \dot{\psi }(x_i,t) = L(\psi (x_i,t)) \\
&& \quad +\, \int _{\Gamma _l} g_{ij}S(\psi (x_j,t-\tau _{ij}))dx_j \\
&& \quad +\, \int _{\Gamma _g} G_{ij} \eta _{ij}S(\psi (x_j,t-\tau _{ij}))dx_j +w(t). \\
\end{eqnarray*}

Let ψ(*x_i_, t*) be the neural activity at time *t* and position *x_i_*. Here *L* describes the local dynamic field as a function of local activity ψ(*x_i_, t*). Local neural activities are also influenced by incoming input through its connections, either from nearby tissue (local connectivity *g_ij_*) or from distant brain regions (global connectivity *G_ij_*). Within the local domain Γ_*l*_, each vertex *i* is connected locally through homogeneous connections. Local connections, described by *g_ij_*, are defined as the geodesic distance along the cortical surface between vertices *i* and *j*. Function *S* is a nonlinear function of ψ at a space point *x* and a time point *t* − τ. The spatial domain Γ_*g*_ is defined on the whole brain and global connections between vertices *x_i_* and *x_j_*, through white matter tracts, weighted by the corresponding element *G_ij_* of the connectivity matrix and delayed by τ_*ij*_. We also introduced the factor η_*ij*_ modulating the connection weight from region *i* to the target region *j*. Although *G_ij_* and η_*ij*_ share mathematically equivalent roles in the equation, they represent different physiological and pathophysiological concepts. The global connection weights *G_ij_* are estimated from white matter fibers, whereas η_*ij*_ represents their pathophysiological modulation. In some brain disorders such as Parkinson’s disease and schizophrenia, anomalies in neurotransmission and the associated pathways can influence communication between certain brain regions, affecting the effective connection weight. This influence is captured by η_*ij*_. In cases where neurotransmitter pathways are not affected, η_*ij*_ = 1. The term *w*(*t*) denotes the dynamical noise. The dynamic range of Equation 2 has been discussed in detail for various forms of local and global connectivity [12]. The set of control parameters {*k*} (see Equation 1a) is comprised in various components of Equation 2 as a function of inter-individual variability, as well as differences between physiological and pathological conditions. For example, the local dynamics is affected in epilepsy and Alzheimer’s disease, such that {*k*} is related to epileptogenicity of local dynamics and to accumulation of amyloid β, respectively; in ageing, Parkinson’s and multiple sclerosis, {*k*} can be related to global connectivity *G_ij_*, connectivity weights or time delay τ_*ij*_; and in psychiatric disorders, {*k*} can be related to both local dynamics and global connectivity weights. We discuss these topics in detail below.

In this article, we describe virtual brain twins and their standard model at two levels of spatial resolution. The first is a detailed, high-resolution model based upon neural fields (as Equation 2 with spatiotemporal dynamics) and a certain degree of biological plausibility. The second features fewer nodes represented by neural masses and a more parsimonious parameterization, which renders it computationally lighter and more suitable for model inversion or fitting. In this case, local connectivity is ignored, *g_ij_* = 0, and assumed to be absorbed in the neural mass dynamics.

### Personalized whole-brain network modeling

Personalized virtual brain modeling relies on subject-specific parameters extracted from an individual’s (typically multimodal) brain imaging data. Three-level personalization introduces the individual’s recorded data into personalized models in three stages. The first level is to build a whole-brain model on the subject-specific brain space, which considers the individual unique brain anatomical structures. One typically constructs the virtual brain twin as a network of regions, with each region represented as a node in the brain network. These regions are usually defined by atlases and the corresponding locations of regions are derived from T1-MRI. The second level is to directly map connectivity and other parameters into the brain models. The connectivity between nodes is inferred from either functional or structural data. Functional connectivity describes the statistical dependencies (such as correlation, coherence, etc.) between measured brain activity signals [24,25], and is thus limited to the measured regions and spatial resolution. Structural connectivity is derived from DW-MRI, which has been extensively used to map white matter tractography in the brain. In the personalized whole-brain network model, we directly map structural connectivity into parameters *G_ij_* of the standard model (Equation 2). In the same way different data modalities can be mapped onto the model, as, for example, PET loadings can be used to inform regional neural parameter variability. The third level is to infer the clinically relevant parameters by model inversion or data fitting. Model inversion uses functional data, in which the choice of data features is important as it will determine the identifiability of the underlying causes parameterized by parameters *k*. A related issue is degeneracy, which is linked to identifiability and more systematically discussed in the Degeneracy subsection below.

Data features summarise valuable information from human brain data and are the primary input into the personalized modeling process. The selection of an appropriate data feature in multimodal data depends on both the purpose of the virtual brain twin and the functional consequence of a hypothetical cause of a disease. For instance, in epilepsy the power envelope of stereotactic electrophysiological data, which characterize seizure generation and propagation patterns, has been successfully used in the estimation of the epileptogenic network [26,27]. For non-invasive epileptogenicity estimation, the spike rate in MEG or EEG can serve as a pertinent data feature [28]. Another example is given by the different data features that can be derived from resting-state fMRI for healthy ageing and schizophrenia. In the context of healthy ageing, not only the static functional connectivity between brain regions is informative, but also the dynamic change of functional connectivity, which is age related, should be included [29]. In schizophrenia, the frequency-specific functional connectivity proves meaningful. A study found differences between patients with schizophrenia and a control group that were most salient in the frequency interval 0.06–0.125 Hz [30].

Parameters can be inferred using probabilistic machine learning and artificial intelligence techniques. Bayesian inference offers a natural way to obtain the posterior distribution of parameters by combining prior knowledge (collected before seeing the data) with information provided by empirical data, through the so-called likelihood function [31,32] (which represents the conditional probability of observed data given parameters and models). As the gold-standard technique, Markov chain Monte Carlo (MCMC) algorithms sample the posterior distribution of model parameters through random simulations and evaluation of the consistency with empirical data [26,33,34]. Bayesian inference using MCMC provides full information about posterior densities rather than a single point estimate (maximum likelihood [35] or maximum a posteriori [36]). This allows for the quantification of uncertainty, hypothesis testing and finding optimal parameter settings while preserving their correlation structure. Adaptive and gradient-based MCMC sampling in automatic tools allows for unbiased and precise estimations [33,34,37], especially in high-dimensional parameter spaces, which is crucial for precision medicine [26,32]. Because of the complexity of personalized brain models and high dimensionality of the data, Bayesian inference using MCMC can be challenging. Indeed, the analytical form of the likelihood function is often unavailable and numerical evaluation is computationally expensive [38]. In such cases, probabilistic machine learning algorithms, such as neural density estimators [38,39], can efficiently estimate the posterior of the parameters given low-dimensional data features. For instance, the simulation-based inference framework [40,41] applies Bayesian inference with the use of deep neural density estimators to efficiently estimate the posterior distribution of model parameters, while reducing the computational challenges for the calculation of likelihood [42,43].

## CLINICAL USE OF VIRTUAL BRAIN TWINS

**Figure 2.**
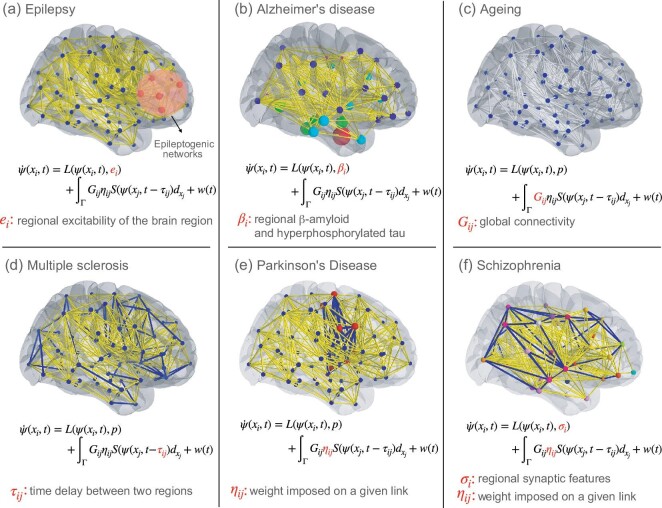
The spatial masks of six clinical uses and their control parameters {*k*} = {*e_i_*, β_*i*_, *G_ij_*, τ_*ij*_, η_*ij*_, σ_*i*_}. (a) In epilepsy, the control parameter set is composed of regional excitability *e_i_* of the local dynamics. The nodes in red with high *e_i_* belong to an epileptogenic network. (b) In Alzheimer’s disease, the control parameter set is composed of the regional parameter β_*i*_ of the local dynamics. The nodes in different colors and sizes show that β_*i*_ depends on amyloid β or tau depositions (Braak stages). (c) In ageing, the control parameter set is composed of the structural connectivity *G_ij_*, illustrated by the network links in white. (d) In multiple sclerosis, the control parameter set is composed of time delays τ_*ij*_. The affected links are colored blue. (e) In Parkinson’s disease, the control parameter set is the link weight η_*ij*_ imposed to link from region *i* to region *j*. The affected links are illustrated in blue and the affected nodes in red represent the basal ganglia-thalamocortical circuit. (f) In schizophrenia, the control parameter set is composed of both the link weight η_*ij*_ and the regional parameter σ_*i*_. The affected links are illustrated in blue and the regional parameter σ_*i*_ in different colors is determined by the balance of excitation and inhibition of region *i*.

Virtual brain twins aim to improve the diagnosis, treatment and prognosis of patients with brain disorders. They can also help to further our understanding of diseases by testing potential pathological mechanisms. Next, we describe the applications of personalized whole-brain network models from a clinical perspective. We start with a mature example: the virtual epileptic patient (VEP) [26,32,44], a personalized whole-brain network model in epilepsy. We then present state-of-art concepts using personalized whole-brain network modeling in Alzheimer’s disease, healthy ageing, multiple sclerosis, Parkinson’s disease and psychiatric disorders. A summary of the parametrization of virtual brain twins for the above clinical uses is shown in Table 1. We introduce the term ‘spatial mask’ to describe a spatial filter imposed on brain networks (nodes or links). A spatial mask is related to the control parameter by capturing its spatial distribution for different brain conditions. Figure 2 presents an overview of spatial masks used in personalized whole-brain models for healthy ageing and five clinical diseases. Potential applications of virtual brain twins may find utility in other clinical domains, such as the brain-computation interface [45] and stroke recovery [46]. These topics are not within the scope of this paper.

**Table 1.** Parametrization of virtual brain twins for clinical uses.

Topic	Hypothesis	Control parameter	{*k*}	Data	Usage
Epilepsy	Excitability	Regional parameter	*e_i_*	T1-MRI, DW-MRI, CT, SEEG, EEG, MEG	Estimation of EZN*, medication, surgery, stimulation
Alzheimer’s disease	Amyloid β, tau	Regional variability	β_*i*_	T1-MRI, PET, DW-MRI, fMRI, EEG, MEG	Stage diagnosis, medication
Ageing	Deteriorated fiber	Structural connectivity	*G_ij_*	T1-MRI, DW-MRI, fMRI	Early diagnosis, neurostimulation
Multiple sclerosis	Slower conduction velocities	Propagation delays	τ_*ij*_	T1-MRI, DW-MRI, EEG, MEG	Clinical monitoring
Parkinson’s disease	Dopamine concentration	Link weights	η_*ij*_	T1-MRI, LFP, PET, DW-MRI, MEG, fMRI	Neurostimulation (tuning)
Schizophrenia	Excitation/inhibition balance, neuromodulatory pathways	Regional parameter, link weights	σ_*i*_, η_*ij*_	T1-MRI, PET, DW-MRI, fMRI, EEG, MEG	Early diagnosis, medication, stimulation

* EZN: epileptogenic zone network.

### Epilepsy

Epilepsy is characterized by recurrent spontaneous seizures that have complex spatiotemporal dynamics involving several connected brain structures and multiple patterns of temporal spread (with changes in frequency, latency and synchrony). Epilepsy affects around 50 million people worldwide and can cause long-term disability. VEP targets patients with drug-resistant focal epilepsy (around 30% of cases) and candidates for surgical treatment as a curative option. Presurgical evaluation is performed to establish whether and how surgical treatment might stop seizures without causing neurological deficits. Precise estimates of epileptogenic networks are crucial for planning intervention strategies.

**Figure 3.**
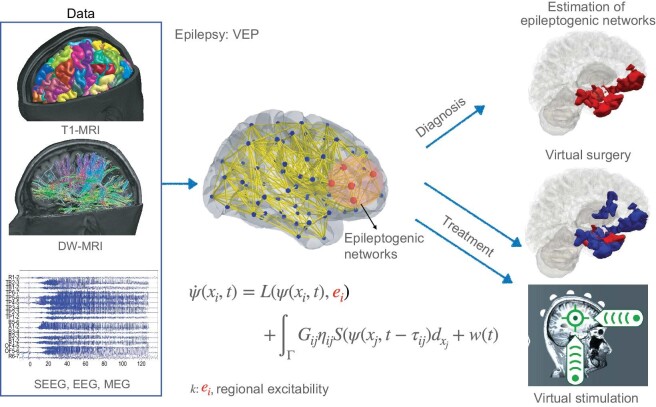
A workflow of a virtual brain twin in epilepsy: virtual epileptic patient (VEP). In the middle is a personalized whole-brain network model, defined by the network of regions. The computational neuronal source activity model in Equation 2 works on each brain region (blue and red spheres) defined by the VEP atlas. The brain regions are connected through the connectome (yellow lines). The brain geometry data from T1-MRI defined distinct brain regions according to the VEP atlas. Tractography was used to estimate the length and density of white matter fibres from DW-MRI (yellow lines in the virtual brain model), which establishes the connectome that specifies the connection strength and time delays via signal propagation between two brain regions. The control parameter {*k*} is the excitability of brain region *e_i_*. The probabilistic machine learning methods are able to obtain the control parameters from SEEG, EEG or MEG. The healthy regions are shown as blue squares and epileptogenic networks as red squares. The VEP can be used for epileptogenic network estimation, virtual surgery and virtual stimulation.

The first version of VEP uses personalized brain models and machine learning methods to estimate epileptogenic networks and to aid surgical strategies [26,32,44] (see Fig. 3). The structural scaffold (162 whole-brain regions and its network) of the patient-specific whole-brain network model is constructed from anatomical T1-MRI and DW-MRI using the VEP atlas [47]. Each network node is equipped with a mathematical dynamical model (defined in Equation 2) to simulate seizure activities. We used the Epileptor [16] to define the local dynamic *L*(ψ(*x_i_, t*), {*k*}) in Equation 2. The control parameter *k* is the excitability *e_i_* on each brain region, for which we define a threshold *e*_θ_. If *e_i_* ≥ *e*_θ_, the corresponding brain region *i* is part of the epileptogenic network (red nodes in Fig. 3) where seizures may start from. The regions with *e_i_* < *e*_θ_ (blue nodes in Fig. 3) are outside the epileptogenic network. The threshold *e*_θ_ is dependent on other personalized parameters such as the structural connectivity. Its spatial mask is highlighted in Fig. 2(a). Probabilistic machine learning methods (such as Hamilton Monte Carlo [33,34] and simulation-based inference algorithms [38]) are used to sample and optimize [36] the control parameters of the personalized model based on functional SEEG recordings of patients’ seizures. These control parameters together with the personalized model determine a given patient’s epileptogenic networks [26]. Personalized models were further used to predict the outcome of surgical interventions using virtual surgeries. We evaluated the VEP workflow retrospectively using 53 patients with drug-resistant focal epilepsy [26,48]. VEP reproduced the clinically defined epileptogenic networks with a precision of 0.6, where the physical distance between epileptogenic regions identified by VEP and the clinically defined epileptogenic networks was small. Compared with the resected brain regions of 25 patients who underwent surgery, VEP showed lower false discovery rates in seizure-free patients (mean of 0.028) than in non-seizure-free patients (mean of 0.407) [26]. VEP is now being evaluated in an ongoing prospective clinical trial (EPINOV: clinical trial identifier NCT03643016) with 356 epileptic patients.

The second generation of VEP uses a high-resolution neural field model (HR-VEP), where the neural models in Equation 2 are built on each of the 260 000 vertices representing the brain with a mm^2^ spatial resolution. The HR-VEP simulates neural activity continuously in space and time, and takes into account the electrical dipole orientation normal to the surface for more accurate mapping of brain activity to the recording sensors such as SEEG, EEG and MEG. For clinical use, we started to seek a non-invasive diagnosis such as the estimation of epileptogenic networks using EEG and MEG, and a non-invasive treatment such as temporal interference stimulation [49] and transcranial direct-current stimulation [50]. We can introduce the stimulation intervention (both invasive, such as SEEG-induced stimulation or deep brain stimulation, and non-invasive) through external input $\hat{u}$ in the standard model (Equation 2). Both VEP and HR-VEP can simulate different stimulation strategies to make virtual stimulation modes, which have capabilities to (1) provide optimized stimulation parameters [51] and (2) predict the stimulation effect(s) at both the local and global scales [52]. The VEP framework has also been further applied to study the dynamics of status epilepticus, and in particular how the propagation depends on the structural connectivity and the global state of the brain network [53].

### Alzheimer’s disease

Alzheimer’s disease is a devastating neurodegenerative disease characterized by a progressive decline in cognitive function. It is estimated that 51 million people worldwide suffered from the disease in 2019 [27] and this number is expected to triple by the year 2050 [54]. This emphasizes the need for further understanding of the disease mechanism and the development of novel therapies. The two pathophysiological hallmarks of the disease are the accumulation of amyloid-β plaques and neurofibrillary tangles of hyperphosphorylated tau protein that lead to neuroinflammation, cell damage and ultimately neuronal death [55]. Recent studies have shown that disease-modifying drugs capable of removing amyloid-β plaques have the potential, even debatable, to decelerate the disease progression [56], indicating the need for an early detection of the disease to maintain cognitive ability. In addition to molecular and cellular pathomechanisms, disease effects can also be observed at the mesoscale circuit and the whole-brain level. On the level of EEG and MEG recordings, a diffuse slowing of the oscillations and altered event-related potentials have been observed [57]. Furthermore, altered spreading of large-scale aperiodic activities is predictive of clinical impairment [58]. In structural MRI, a precise pattern of atrophy was reported [59] and also in fMRI disconnections in resting-state networks [60].

Brain network models have already been used to explain and predict the spatiotemporal propagation pattern of misfolded amyloid-β [61] and tau proteins [62] along the disease trajectory. Furthermore, brain network models have been used to test the link between the patient-specific amyloid-β plaque distribution, hyper-excitation and slowing of neural oscillations [63] in patients with Alzheimer’s disease. The simulated features of the brain network model have then been used to improve classification performance between patients with Alzheimer’s disease, mild cognitive impairment or healthy controls [64]. As mentioned, the goal of brain network models is to further our understanding of the pathomechanism of the disease, to bridge the gap between structural and functional data and to make predictions for the individual patient. In the case of Alzheimer’s disease, we define the regional variability β_*i*_ as the control parameter *k* in the standard model (Equation 2). The regional variability β_*i*_ is related to the spatial pattern of atrophy, amyloid-β or tau depositions (Braak stages) for each region. The spatial mask of Alzheimer’s disease is illustrated in Fig. 2(b). The parameter β_*i*_ reflects the pathophysiological process induced by amyloid β or tau within the local model that is used to represent neural activity in node *i* of the network, which links to parameters of increased neural excitability or decreased inhibitory function [65]. Estimating each control parameter involves utilizing patient-specific data and informed constraints derived from biology [66]. Imaging data used to infer and estimate control parameters could be fMRI and tau–amyloid-β PET [67], a combination of DW-MRI and tau–amyloid-β PET [68], and a combination of amyloid-β, tau and fluorodeoxyglucose PET [69]. Frequency features and functional connectivity are estimated from EEG and MEG functional recordings [70] and complexity analysis from EEG, MEG and fMRI [71].

Both structural connectivity and functional connectivity are linked to the patterns of amyloid-β and tau accumulation and spread [72,73]. Connectomes derived from patient-specific DW-MRI can be integrated into a personalized model, aiding in predicting the patient’s cognition [74] and facilitating a better understanding of the roles of connectivity in the progression of Alzheimer’s disease pathology. Network connectivity plays a vital role in various brain orders, such as epilepsy, Alzheimer’s disease and schizophrenia, by influencing the brain dynamics; however, it is not labeled as a control parameter here because it is not specific to one disease.

### Ageing

Healthy ageing is accompanied by a decline of cognitive abilities with substantial variations among the individual ageing trajectories, in particular at later stages in life [75,76]. Many studies have shown that this variability is associated with the organizational changes of ageing in both structural [77,78] and functional connectivity [29,79], but without testing the possible causality between the two. Fiber connections are expected to deteriorate [80], particularly with respect to the number of inter-hemispheric fibers within tracts and fiber density [42,81]. In addition, time delays due to white-matter propagation are also affected by demyelination during ageing [82,83]. Time delays have been shown to support age-related functional alterations in the human brain [81], together with the dynamical compensation for the white-matter degradation, in particular causally linked to interhemispheric white-matter degradation in the virtual ageing brain (VAB) framework [42]. There, we built a personalized whole-brain network model and defined *G_ij_* to be the control parameter *k* in the standard model (Equation 2), illustrated in Fig. 2(c). The structural connectivity can be directly mapped from subject-specific DW-MRI data. In VAB, a mask applied to interhemispheric connections of a younger subject is used to reproduce the process of functional dedifferentiation during ageing. By virtually ageing younger and older subjects, the VAB showed that the decrease in fluidity of functional connectivity dynamics with age is likely driven by interhemispheric white-matter degradation. VAB was also able to predict cognitive performance in older adults, using simulation-based inference [42] to estimate the scaling of *G_ij_*. The VAB thus offered the first direct evidence of dedifferentiation in ageing leading to adverse effects of cognitive decline in a large cohort.

The virtual brain twin of a healthy ageing brain can also help identify brain states and early signatures of brain disorders and then develop possible interventions such as neurostimulation to improve declining cognitive functions. Another study [84] uses a whole-brain computation model to simulate fMRI activity and predict functional connectivity. Based on longitudinal studies that have shown an association between altered resting-state functional connectivity and decreased cognitive functions [85,86], this study [84] shows that *in silico* stimulation of each node was able to induce transitions from the brain state profile of the older- to the middle-aged group. They found that the precuneus was the best stimulation target to achieve this functional reconfiguration.

### Multiple sclerosis

Multiple sclerosis is a chronic, autoimmune and degenerative disease of the central nervous system that affected a total of 2.8 million people worldwide in 2020 [87]. The immune system attacks the myelin sheath, which coats nerve fibers (axons) and supports saltatory conduction, responsible for various motor and cognitive symptoms. Virtual brain twins might be particularly useful for patient stratification (given the heterogeneous nature of the disease) as well as for predicting the effect of therapeutic changes (e.g. therapy switch). The increasing number of treatment options on the one hand, and the availability of large multimodal datasets on the other hand, bear promises for the deployment of personalized models in the near future [4]. The existing prediction models have investigated the individual response of patients with multiple sclerosis to disease-modifying therapies, using generalized linear models [88–90]. These studies aimed to predict individual clinical responses from large, multidimensional datasets. However, the models did not attempt a direct mechanistic account of the emergence of patient-specific clinical disabilities [4].

Here, we introduce a personalized whole-brain network model for patients with multiple sclerosis. The rationale of this approach is based on the idea that symptoms in multiple sclerosis are caused by slower conduction velocities. These cannot be directly measured across the whole brain and, as a consequence, structural lesions are typically used to assess damage accumulation. The virtual brain twins might allow us to directly infer the conduction velocities. In fact, multiple sclerosis patients demonstrate greater functional delays across the whole brain, as compared to healthy subjects, and all the more so for tracts affected by structural lesions [91]. Also, changes in myelination can alter the timing of the interactions among brain regions [78], thus leading to symptoms. Thus, the control parameter *k* for this disease is the time delay τ_*ij*_ as defined in the standard model (Equation 2), and its spatial masks are shown in Fig. 2(d). The time delay τ_*ij*_ in virtual brain twins can be inferred from a functional data feature such as the power spectrum density of patient’s MEG recordings, while structural connectivity *G_ij_* is inferred from DW-MRI recordings directly. Furthermore, virtual brain twins can predict clinical disability and the activity of pathophysiological mechanisms by inferring subject-specific conduction delays.

### Parkinson’s disease

Parkinson’s disease is the second most common neurodegenerative disease causing motor symptoms such as tremor, rigidity and bradykinesia, as well as other non-motor symptoms [92]. The prevalence of Parkinson’s disease is strongly age dependent, ranging from 0.04% to 2% in the age groups 40–49 and older than 80, respectively [93]. The pathological hallmark of Parkinson’s disease is the accumulation of misfolded α synuclein in Lewy bodies and the degeneration of dopamine-producing neurons in the substantia nigra [92]. Loss of dopaminergic nigrostriatal neurons along the nigrostriatal pathways and a more modest loss along the mesolimbic and mesocortical can be implemented in a personalized whole-brain network model by introducing η_*ij*_—a spatial mask modulating the connectivity weight between region *i*, the source of the neuromodulator, and region *j*, the target. The link weights η_*ij*_ can be considered as control parameter *k*. Anomalies in neurotransmitter pathways can influence communication between brain regions, while the white matter fibers connecting these regions remain unaffected. The spatial mask in Parkinson’s disease is shown in Fig. 2(e), where the affected links are illustrated in blue and the affected nodes in red represent the basal ganglia-thalamocortical circuit. The control parameter can be mapped and inferred from a patient’s specific invasive and/or non-invasive recordings. Some studies combine invasive and non-invasive modalities, for example, MEG is combined with subthalamic local field potential recordings [94,95] and fMRI with deep brain stimulations [96]. The connection between the cortex and subthalamic nucleus can also be investigated using simultaneous 18F-FDG-PET and fMRI [97].

In addition to the molecular and cellular pathological mechanisms, larger-scale dynamical phenomena are observed. Altered dynamics in the basal ganglia causes anomalous bursts of activities in the beta frequency range, which are related to the clinical disability [98]. The presence of stereotyped large-scale dynamics has also been linked to clinical disability [99]. Deep brain stimulation in subcortical nuclei is used to ‘desynchronize’ neural activity and thus improve the symptoms [100]. Computational modeling studies have investigated this phenomenon in neural network models [101,102] as well as in basal ganglia-thalamocortical circuit models [103], in which pathological oscillations may arise due to altered connectivity. Brain network models can be used to predict the optimal stimulation paradigm in silico, represented by *u* in our standard model. Additionally, the brain network can help in distinguishing different kinds of Parkinsonism. A recent study has shown that complementing empirical functional connectivity of fMRI recordings with simulated data from patient-specific whole-brain network models could enhance the classification of Parkinson’s patients [104]. Another computational model investigated changes in the basal ganglia pathway inferred from resting-state fMRI [105].

### Psychiatric disorders

Psychiatric disorders are a heterogeneous group of disorders, characterized by a clinically significant disturbance in an individual’s cognition, emotional regulation or behaviour. According to a report from the World Health Organization, psychiatric disorders affected 970 million people around the world in 2019 before COVID-19 [106]. To date, most psychiatric disorders lack precise biomarkers, and their main pathophysiological hypotheses are still debated [107,108]. In the case of schizophrenia, affecting approximately 24 million people worldwide, the classical hypothesis involves a dysfunction in neurotransmission and neuromodulation. Low dopamine levels within the mesolimbic pathway, extending from the ventral tegmental area to the limbic areas, are thought to be responsible for positive psychotic symptoms [109]. Low dopamine levels within the mesocortical pathways, which extend from the ventral tegmental area to the cortex, are thought to cause the negative symptoms and cognitive deficits [109]. The neuromodulatory pathways can be implemented via the spatial mask η_*ij*_ modulating the connectivity weight between region *i*, the source of the neuromodulator, and region *j*, the target. The spatial mask η_*ij*_ can be formalized as a system variable changing in time according to the fluctuations of dopamine release and its impairments. Further evidence shows a disruption of the cortical excitation/inhibition balance, whether it is through synaptic pruning [110] or under the effect of gamma-aminobutyric acid transmission or of N-methyl-D-aspartate receptor plasticity [111]. These changes in local dynamics are introduced by region-specific parameters σ_*i*_ that can represent the balance between excitation and inhibition in region *i* or even synaptic density. Parameters σ_*i*_ plus η_*ij*_ become the control parameters {*k*} of the standard model (Equation 2), and its spatial mask is shown in Fig. 2(f). These two subsets of control parameters can be mapped directly and inferred from personal structural and functional recordings. MRI recordings have shown impaired gyral formation in the anterior cingulate cortex [112] and the frontal cortex [113] in patients with schizophrenia. MRI recordings have also provided evidence that schizophrenia is associated with lower gray matter volumes, in particular in the frontal cortex [114]. In schizophrenia patients, [11C] UCB-J PET imaging shows significantly lower synaptic vesicle protein 2A density in frontal and anterior cingulate cortices, indicating lower synaptic density [115]. Genetic studies [110], EEG, MEG [116] and fMRI [117] have provided evidence for altered E/I balance in schizophrenia. These patient-specific in vivo recordings can provide the input data for training virtual brain twins in schizophrenia.

Other studies using multiregion network modeling demonstrate consistently increased self-inhibition in frontal areas and dis-inhibition in auditory areas in schizophrenia. Here the computational model contains six brain regions, each of which includes pyramidal, spinal stellate cells and inhibitory interneurons [118]. The local parameters and regional connections are inferred and compared using dynamic causal modeling based on EEG and fMRI features. Similar approaches, not exclusive to schizophrenia, have already been experimented in several studies, to explore the dysconnection hypothesis [119,120], the neuromodulatory effect of a psychoactive drug on brain function in healthy subjects [121] or in a stimulation paradigm [122]. The advent of multiscale brain models will add further granularity to the control parameters and bring hope in disentangling the complex interplay of mechanisms responsible for the emergence of psychiatric disorders.

## KEY CHALLENGES AND FUTURE DIRECTIONS

Given all the existing and envisioned clinical use cases explained above, there are certain aspects and possible pitfalls that are common to any application of virtual brain twins. In the following subsections we highlight those challenges, such as degeneracy and overfitting, and we also give an outlook for future model improvement using high-resolution, co-simulation and deep generative models.

### Degeneracy

Degeneracy refers to the ability of structurally different elements to produce the same function or behavior, and is a natural property of the brain [123–125] due to its multiscale nature. From a biological perspective, degeneracy underpins the resilience of the brain. However, from an inference perspective, identifying and disentangling degeneracy is a significant challenge, as it involves deciphering how various model configurations can lead to similar functional outcomes [126], associated with an increase in computational cost. Challenges regarding identifiability in inference thus arise from two aspects: (1) insufficient data or oversimplified models and (2) degeneracy. The first aspect is technical and can be addressed by incorporating additional information such as multimodal imaging data, integrating multiscale models and co-simulation, and introducing reparameterization techniques within the model configuration space. The coexistence of models with different levels of description from the most biophysically detailed to the most phenomenological may also help to address this challenge [127]. The second aspect is intrinsic to the brain and needs to be conceptually integrated and dealt with as an important brain characteristic when we design personalized brain models and make interpretations in clinical use [26].

### Overfitting versus precision medicine

Precision medicine can be defined as an approach that delivers the effective treatments to a specific patient at the optimal time [128]. The complex biological disease needs to be reduced to its components from which the most relevant features can be identified and measured to choose an optimal intervention [129]. When these measures greatly outnumber the amount of samples they are made on, there is a risk of overfitting. When a model is fitted to a specific dataset, known as the ‘training’ set, it may perform exceptionally well, to the extent that it captures detailed but irrelevant features, such as noise. This causes accuracy issues when a new dataset is introduced (the ‘test’ set), because the model cannot generalize to unseen data [129]. Model regularization techniques are a common approach for dealing with such issues. For instance, imposing constraints (such as connectivity) on the model complexity can effectively prevent overfitting and enhance the model’s generalizability, making them reliable in a probabilistic data analysis (and eventually in a multicentric context). Traditional evidence-based medicine relies on randomized clinical trials in order to determine the most effective treatment for a particular class of patients. The challenge of precision medicine is that of making predictions based on subject-specific features without losing clinical relevance in nosographic terms.

### Crossing multiscale models and co-simulation

Scale integration in computational brain modeling involves bridging various levels of complexity, from single neurons to whole-brain models. On the one hand, the bottom-up approach begins with models capturing single neuron excitability, serving as fundamental building blocks for spiking neural networks. Network complexity can be managed through mean-field approaches, which simplify large-scale network dynamics by considering average population behavior. These mean fields thus represent neural masses. With respect to the whole-brain perspective, each brain region is represented by a neural mass model. On the other hand, the top-down approach may help identify possible minimal mechanisms needed for the emergence of whole-brain dynamics. The co-contribution of these approaches helps build relevant models for specific digital twin applications. Once the whole-brain network is built, one or multiple regions can be replaced by their corresponding lower-scale model. In such a case, known as co-simulation, a neural mass is replaced by the corresponding spiking neural network. It allows testing specific hypotheses about cross-scale communication, between the cellular or sub-cellular scale (for a region of interest) and the whole-brain level. Methods based on machine learning techniques (such as neural ordinary differential equations [130] and polynomial regression) can also be used to learn the behavior of microcircuit models for which we lack mean-field derivations. These learned parametric manifolds of neuronal activity can then be transferred to a whole-brain network model, allowing for the traversing of scales.

### Deep generative models for prediction on brain disorders

Generative models are unsupervised machine learning algorithms that learn complex data distributions and generate new data samples, often in the form of images, texts or other structured data. The deep generative models are neural network-based algorithms that are primed to predict the occurrence, progression or outcomes of neurological or psychiatric conditions by learning from relevant data, enabling potential applications in diagnosis, prognosis and treatment planning. Normalizing flows [131,132], which are employed to transform a simple probability distribution into a complex one, have proven to be effective and efficient for probabilistic inference, in epilepsy [38], ageing [42] and focal interventions (chemogenetics and lesions) [43]. Recently, variational autoencoders for nonlinear dynamical system identification demonstrated their capacity to infer both the neural mass model and the region- and subject-specific parameters from the functional data, while respecting the known network structure [133,134]. In the near future, these advanced models can be harnessed for precise, accurate and end-to-end automatic inference on brain diseases from big and multimodal data, with different spatio-temporal scales.

### High resolution

Most of the clinical applications we discussed made use of virtual brain twins at low resolution (∼10 cm^2^ per brain region). In theory, representations of the standard model (Equation 2) at high spatial resolution (∼1 mm^2^) can significantly improve the simulation and predictive power. The change from low to high resolution can increase the precision of source-to-sensor mapping, and also allows for complex intra-regional dynamics with consequences for the overall network organization. In practice, only high-resolution models can simulate some empirically observed signals such as the traveling waves along the cortical surface [135], and guarantee high fidelity of electromagnetic fields in brain stimulation. The control parameters and spatial masks remain the same in each clinical use case, when changing from low to high resolution. Our ongoing work has shown the application of high-resolution simulation [26] and feasibility of high-resolution model inversion in epilepsy. In the near future, the personalized whole-brain network models in high-resolution can be extended to other clinical use cases.

## CONCLUSION

Virtual brain twins are computational models of human brains that are informed by subject-specific data, enabling individual prediction of neural parameters and interventions. We gave an overview and perspective of the current and possible future use of virtual brain twins for clinical applications. We focused on the process of personalization and identified the control parameters and spatial masks in each clinical use. Currently, the furthest developed example is the VEP for epilepsy. Future development should tackle the challenges of model degeneracy and overfitting, as well as seek to improve accuracy through higher-resolution and multiscale models.
